# Noninvasive Assessment of Gene Transfer and Expression by *In Vivo* Functional and Morphologic Imaging in a Rabbit Tumor Model

**DOI:** 10.1371/journal.pone.0062371

**Published:** 2013-06-10

**Authors:** Murali K. Ravoori, Lin Han, Sheela P. Singh, Katherine Dixon, Jyoti Duggal, Ping Liu, Rajesh Uthamanthil, Sanjay Gupta, Kenneth C. Wright, Vikas Kundra

**Affiliations:** 1 Department of Experimental Diagnostic Imaging, The University of Texas MD Anderson Cancer Center, Houston, Texas, United States of America; 2 Department of Diagnostic Radiology (Section of Body Imaging), The University of Texas MD Anderson Cancer Center, Houston, Texas, United States of America; 3 Department of Diagnostic Radiology (Section of Interventional Radiology), The University of Texas MD Anderson Cancer Center, Houston, Texas United States of America; 4 Department of Biostatistics, The University of Texas MD Anderson Cancer Center, Houston, Texas, United States of America; 5 Department of Veterinary Medicine and Surgery, The University of Texas MD Anderson Cancer Center Houston, Texas, United States of America; AMS Biotechnology, United Kingdom

## Abstract

**Purpose:**

To evaluate the importance of morphology in quantifying expression after *in vivo* gene transfer and to compare gene expression after intra-arterial (IA) and intra-tumoral (IT) delivery of adenovirus expressing a SSTR2-based reporter gene in a large animal tumor model.

**Materials and Methods:**

Tumor directed IA or IT delivery of adenovirus containing a human somatostatin receptor type 2A (Ad-CMV-HA-SSTR2A) gene chimera or control adenovirus (Ad-CMV-GFP) was performed in VX2 tumors growing in both rabbit thighs. Three days later, ^111^In-octreotide was administered intravenously after CT imaging using a clinical scanner. ^111^In-octreotide uptake in tumors was evaluated the following day using a clinical gamma-camera. Gene expression was normalized to tumor weight with and without necrosis. This procedure was repeated on nine additional rabbits to investigate longitudinal gene expression both 5 days and 2 weeks after adenovirus delivery. CT images were used to evaluate tumor morphology and excised tissue samples were analyzed to determine ^111^In-octreotide biodistribution ex vivo.

**Results:**

VX2 tumors infected with Ad-CMV-HA-SSTR2 had greater ^111^In-octreotide uptake than with control virus (*P*<0.05). Intra-arterial and intra-tumoral routes resulted in similar levels of gene expression. Longitudinally, expression appeared to wane at 2 weeks versus 5 days after delivery. Areas of necrosis did not demonstrate significant uptake *ex vivo*. Morphology identified areas of necrosis on contrast enhanced CT and upon excluding necrosis, *in vivo* biodistribution analysis resulted in greater percent injected dose per gram (*P*<0.01) and corresponded better with *ex vivo* biodistribution(r = 0.72, *P*<0.01, Coefficient of the x-variable = .72) at 2 weeks than without excluding necrosis (*P*<0.01).

**Conclusion:**

Tumor specificity and high transgene expression can be achieved in tumors via both tumor directed intra-arterial and intra-tumoral delivery in a large animal tumor model. Using clinical machines, morphologic imaging contributes to functional imaging for quantifying SSTR2-based reporter expression *in vivo*.

## Introduction

Gene therapy can be defined as the introduction of genetic material into cells for a therapeutic purpose [1]. For *in vivo* gene therapy, the gene medicine formulation is commonly injected directly into the patient. Although there have been a few reports where clinical efficacy [2]–[6] or even complete cure [7] has been demonstrated, clinical trials are significantly hampered by a lack of clinically relevant methods for *in vivo* detection of gene transfer [8]. Currently in the clinic, evaluating success of gene transfer is primarily limited to analyses of biopsy samples, yet the information collected from this approach is restricted to a few cubic millimeters of biopsy material. As a result, this method provides limited assessment of *in vivo* gene delivery, is prone to sampling error, has associated morbidity and mortality, and can have problems with patient compliance especially when repeated evaluation is needed.

Instead, monitoring of exogenous gene expression should be noninvasive and easily repeatable over time in the same patient. This would inform regarding the location, magnitude, and kinetics of gene expression, and, could prove instrumental towards the rational development of innovative formulations designed to selectively target particular tissues, organs, or disease sites. To approach these needs, reporter genes may be used. These often encode enzymes, transport proteins, and receptors [9]–[13] that most frequently bind and/or entrap an imaging agent.

Radiolabelled somatostatin analogues, such as ^111^In-octreotide, are commonly used in patients for the detection of rare neuroendocrine tumors expressing the human somatostatin receptor type 2 (SSTR2). Reporters based on SSTR2 have been proposed [14]–[16]. Recently, we demonstrated *in vivo* imaging of HA-SSTR2 expression after intra-tumoral adenovirus (Ad-CMV-HA-SSTR2) infection in subcutaneous and intrathoracic mouse models [14], [17], [18]. Limitations of mouse models include small tumor and vessel size, which limit approximation of human tumor morphology and vascular access. Large animal models, such as rabbits, overcome these limitations and should allow comparisons such as expression after intra-arterial (IA) versus intra-tumoral (IT) gene delivery.

VX2 tumors are transplantable in rabbits and tend to necrose when they become larger, similar to many human tumors in absence of or due to therapy. For the quantification of gene expression, necrosis is problematic because these “dead” areas can neither express a gene nor can be well distinguished from living tissue using functional imaging; thus, necrosis theoretically may lower apparent expression. A solution may be using morphologic assessment of anatomic images. Unlike mouse models, where cognate human imaging machines are used, larger animal models can be imaged using identical human instrumentation, and serve as an important bridge to translation. In this study, we compare gene expression in tumors after intra-arterial and intra-tumoral delivery of a SSTR2-based reporter gene using a rabbit VX2 tumor model and evaluate the importance of morphology on quantification of gene expression after *in vivo* gene transfer.

## Materials and Methods

### Construction of pAd-HA-SSTR2

Ad-CMV-HA-SSTR2 was constructed as previously described [18]. A large-scale purification of the Ad-CMV-HA-SSTR2 and the control adenovirus containing a CMV promoter with green fluorescent protein (Ad-CMV-GFP) was performed by the Vector Core Laboratory at M.D. Anderson Cancer Center.

### Tumor Model

The animal experiments were performed in accordance with the Guide for the Care and the Use of Laboratory Animals, as approved by the National Research Council and by the Institutional Animal Care and Use Committee at U.T.-M.D. Anderson Cancer Center. The rabbit VX2 adenosquamous carcinoma model was used for the current study [19]. VX2 is a malignant rabbit squamous cell carcinoma [20], [21]. It possesses characteristics similar to human squamous cell carcinomas, including heparin-dependent angiogenesis [22], a biochemical phenotype characteristic of advanced stage tumors (i.e., high glycolysis and elevated levels of mitochondrial bound hexokinase) [23], , and lack of spontaneous regression. VX2 tumor vessels lack smooth muscle [25] like most human tumor angiogenic vessels. Although the VX2 is a transplantable, highly malignant neoplasm of rabbits, VX2 cells are very difficult to grow in culture and must be maintained by serial transplantation in carrier rabbits [26]. We found that we could harvest and freeze tumors, yet maintain viability.

For the present study, the tumors were first sustained by injecting several carrier rabbits with freshly harvested and prepared VX2 tumor fragments in the muscles of each thigh. When the resulting tumors reached approximately 2 cm in diameter, the donor was sacrificed with an overdose of Beuthanasia-D (1 mL/4.5 kg) (Schering-Plough Animal Health Corp., Kenilworth, NJ), administered through a marginal ear vein. The tumors were immediately harvested under sterile conditions, minced, and homogenized in DMEM/F12 containing 10% FBS (Media Tech. Inc., Manassas, VA) and gentamicin. The tumor fragments were aliquoted into 12×75 mm plastic tubes and stored at −80°C until further use. When needed, they were thawed, suspended in DMEM/F12 containing 10% FBS and gentamicin, and drawn into a 1-cc syringe with an 18-gauge needle. A total of 0.6 mL of VX2 tumor suspension was then injected at a single site in both thighs of each experimental rabbit. The procedure resulted in relatively predictable tumor growth.

### Imaging and Biodistribution

Nine adult New Zealand white rabbits (3–4 kg) were used to assess reporter gene expression in VX2 tumors after tumor directed intra-arterial or intra-tumoral *in vivo* gene transfer. In this work we focused on tumor directed IA and IT adenovirus delivery and not systemic intravenous delivery, which has been previously shown to result in expression primarily in the liver [27]–[29]. Banked VX2 tumor fragments were injected intramuscularly in both thighs of each animal (18 tumors total). Ten days later, multi-slice helical CT (Lightspeed Plus; GE Medical Systems, Milwaukee, WI) imaging both without and with contrast enhancement was performed while the animals were in the supine position in order to document VX2 tumor formation and to calculate tumor volume (V) and weight. All images were acquired using a 120-kVp tube voltage, an 80 mA tube current, a 25 cm field of view, and 1.25 mm slice thickness. For contrast-enhanced CT imaging, 8.0 mL of Visipaque 320 contrast medium (iodixanol; GE Healthcare, Inc., Princeton, NJ) was injected at a rate of 1.5 mL/sec through a marginal ear vein. After a 9 second delay, CT images of the thighs were obtained. Similar sized tumors were evenly distributed among three groups. Twelve tumors received Ad-CMV-HA-SSTR2 (100 µL with MOI of 10) via the IA (n = 6) or the IT (n = 6) route, and the other six tumors received an IT injection of Ad-CMV-GFP (100 µL with MOI of 10). Three days later, CT imaging was repeated and the animals were injected via a marginal ear vein with 22 MBq (600 µCi) of ^111^In-octreotide (Mallinckrodt, St. Louis, MO). The following day, planar gamma camera imaging was performed. The amount of ^111^In-octreotide in each tumor was determined from the planar images and normalized to the tumor weight (% ID/g) calculated from the CT images.

After the imaging session, a blood sample from each animal was collected and the rabbits were sacrificed with an intravenous overdose of Beuthanasia-D. Portions of tumor, including areas of live tumor and areas of necrosis, and various normal organs (heart, lungs, liver, spleen, and intestines) were harvested and weighed; the amount of radioactivity associated with the blood and each tissue sample was measured by a γ-counter to determine ^111^In-octreotide biodistribution in % ID/g.

Another nine adult New Zealand white rabbits (3–4 kg) were used to assess gene expression longitudinally. Animals were prepared as described above. After CT imaging, tumors were injected with 100 µL (MOI of 10) of either Ad-CMV-HA-SSTR2 (six IA and six IT) or Ad-CMV-GFP (three IA and three IT). Two rabbits died during the study; one died from carotid artery rupture during catheterization, and the other died during the administration of general anesthesia. As a result of these deaths, the number of tumors, from which imaging and biodistribution data were obtained, was modified as follows: six with IA Ad-CMV-HA-SSTR2, four with IT Ad-CMV-HA-SSTR2, two with IA Ad-CMV-GFP, and two with IT Ad-CMV-GFP. Four days after virus injection, CT imaging was repeated and the animals were injected with 22 MBq (600 µCi) of ^111^In-octreotide via a marginal ear vein. Planar gamma camera imaging was performed the next day. Approximately two weeks later, CT imaging was repeated and the animals were re-injected with 22 MBq (600 µCi) of ^111^In-octreotide. Planar gamma camera imaging was performed again the next day. The amount of ^111^In-octreotide in each tumor was determined from the planar images at each time point and normalized to the tumor weights ± necrosis (%ID/g) calculated from the CT images. Afterwards, tissues were processed for *ex vivo* biodistribution analysis as above.

### Intra-arterial (IA) and Intra-tumoral (IT) injection routes

For IA gene delivery, each rabbit was anesthetized by isoflurane (5%)/oxygen (1.5 L/min) via mask administration. An endotracheal tube was inserted and anesthesia was maintained with isoflurane (3–4%)/oxygen (1.5 L/min). An antibiotic, enrofloxacin (Baytril; Bayer Corp; Agriculture Division, Animal Health, Shawnee Mission, KS) was given at 5 mg/kg intramuscularly, and the neck was shaved and prepared for aseptic surgery using alcohol and betadine scrub. A small midline incision was made and the right common carotid artery was isolated and ligated cranially. A small arteriotomy was made proximal to the ligation, a 4.0 Fr introducer sheath (Cook Incorporated, Bloomington, IN) was inserted into the vessel through the arteriotomy, and sodium heparin (100 IU/kg) was administered through the sheath.

A 2.8 Fr microcatheter (EmboCath; BioSphere Medical, Rockland, MA) was inserted through the sheath and directed down the aorta to the level of the aortic bifurcation under fluoroscopic monitoring. The catheter was then manipulated into each external iliac artery and digital subtraction arteriography was performed to document vascular anatomy as well as tumor location. This was accomplished by hand-injecting radiographic contrast medium (meglumine-diatrizoate) under fluoroscopy. The microcatheter was then positioned into the deep femoral artery, the main, if not exclusive, artery supplying the tumor. The catheter was flushed with saline and 100 µL (multiplicity of infection, MOI, of 10) of adenovirus was slowly injected; the catheter was flushed once again with saline. The catheter and introducer sheath were removed, the carotid artery was ligated proximal to the arteriotomy, and the incision was closed in two layers. The rabbit was then allowed to recover from anesthesia.

For direct IT injection, each rabbit was anesthetized and intubated as described above. An antibiotic (enrofloxacin; 5 mg/kg) was given intramuscularly and the animal was placed in the CT scanner. Using CT image guidance and an 18-gauge needle, 100 µL (MOI of 10) of adenovirus was delivered directly into the tumor at two locations.

### Determination of Tumor Volume and Weights

A region of interest (ROI) was drawn around the whole tumor on the CT images with or without intravenous contrast enhancement. Tumor volume measurements were then performed as previously described [30] using volume viewer application software for CT (Voxtool 3.0.64z, GE Medical Systems). Assuming a tumor density of 1.0 g/mL, tumor volume (cm^3^) was converted to weight [30]–[34]. A similar process was used to trace and calculate the weight of necrotic tissue within the tumor images that were identified as areas of tumor that did not enhance after intravenous contrast. The weight of the necrotic tissue was then subtracted from the tumor weight to determine the weight of the tumor without necrosis.

### Planar Gamma Camera Imaging

Rabbit thighs were positioned 2 cm directly below a medium-energy parallel-hole collimator and underwent planar imaging for 30 minutes with a clinical flexible single-head gamma camera (Digirad Corporation, Poway, CA). The camera and thighs were positioned so that both thighs could be imaged within the 20×20 field of view of the camera. Images were transferred in DICOM format to an eSoft workstation and processed. ROIs of the same size were obtained from the tumor and background regions to calculate average counts per pixel based on imaging of a phantom with various amounts of ^111^In-octreotide in a total volume of 500 µL [30].

### Western Blotting

Protein derived from tumors infected IA or IT with Ad-CMV-HA-SSTR2 or Ad-GFP control was used for performing Western blots as previously described [30] with rabbit-anti-SSTR2A (Santa Cruz, Santa Cruz, CA) at a 1∶3000 dilution and secondary HRP goat anti-rabbit antibody (BioRad, Hercules, CA) at 1∶4000 dilution.

### Statistical Analysis

Results are presented as means ± standard deviation. Wilcoxon rank-sum test was used for making comparisons between groups. P≤0.05 was considered a significant difference. Pearson's correlation was performed between *in vivo* and *ex vivo*-derived weights and biodistribution analyses. Linear regression models were used to compare correlation slopes.

## Results

### Assessment of Reporter Gene (Ad-CMV-HA-SSTR2) Expression in VX2 tumors after In Vivo Gene Transfer

**Figure 1:**
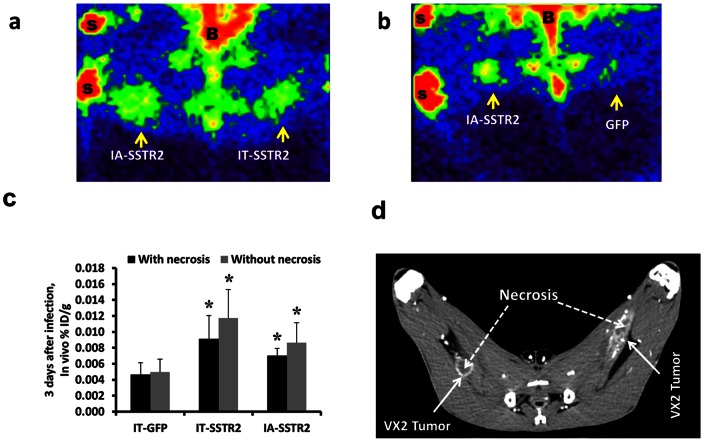
Imaging data of VX2 tumors in rabbits. (a and b) Representative gamma camera planar images of VX2 tumors (a) infected *in vivo* by IA and IT administration of Ad-CMV-HA-SSTR2, and (b) infected *in vivo* by IA infusion of Ad-CMV-HA-SSTR2 and by IT injection of control Ad-CMV-GFP (B =  bladder, S =  source of ^111^In for positioning). Increased ^111^In-octreotide uptake is seen in tumors infected with Ad-CMV-HA-SSTR2 by both routes of administration compared to infection with the control Ad-CMV-GFP. (c) ^111^In-octreotide biodistribution in tumors normalized to tumor weight (%ID/g) calculated with and without necrosis using imaging only (*in vivo* biodistribution from gamma camera and CT imaging). Uptake was higher in tumors infected with Ad-CMV-HA-SSTR2 compared to control Ad-CMV-GFP (*, p<0.01, n = 6 for IA vs GFP; p<0.01, n = 6 for IT vs GFP). No difference was seen between the IA and IT groups. (d) Contrast enhanced CT showing the small amount of necrosis within the tumors.

Inoculation of thawed VX2 tumor fragments resulted in 100% tumor formation. Three days after adenovirus infection, gamma camera imaging demonstrated HA-SSTR2 expression after IA and IT Ad-CMV-HA-SSTR2 delivery (Fig 1A). Fig. 1B also demonstrates HA-SSTR2 expression after IA Ad-CMV-HA-SSTR2 delivery; in contrast, only background signal is seen in the tumor in the opposite leg infected IT with control Ad-CMV-GFP. Confirming the visual results, significantly greater uptake of ^111^In-octreotide was seen in VX2 tumors exposed to Ad-CMV-HA-SSTR2 by either the tumor directed IA or IT routes as compared to the tumors infected with the control Ad-CMV-GFP virus (p<0.01 for IA, n = 6; p<0.01 for IT, n = 6; Fig. 1A, B, & C). Similar findings were noted when the *in vivo* biodistribution was normalized for necrosis (p = 0.01 for IA, n = 6, p<0.01 for IT, n = 6; Fig. 1C). Both tumor directed IA and IT routes of Ad-CMV-HA-SSTR2 delivery resulted in similar expression of the reporter gene. In these small tumors with relatively little necrosis (Fig. 1D), no difference in gene expression was noted within groups when the % ID/g was normalized to CT-determined tumor weight with or without necrosis (Fig. 1C & D).

#### *Ex vivo* findings confirmed in vivo results

**Figure 2:**
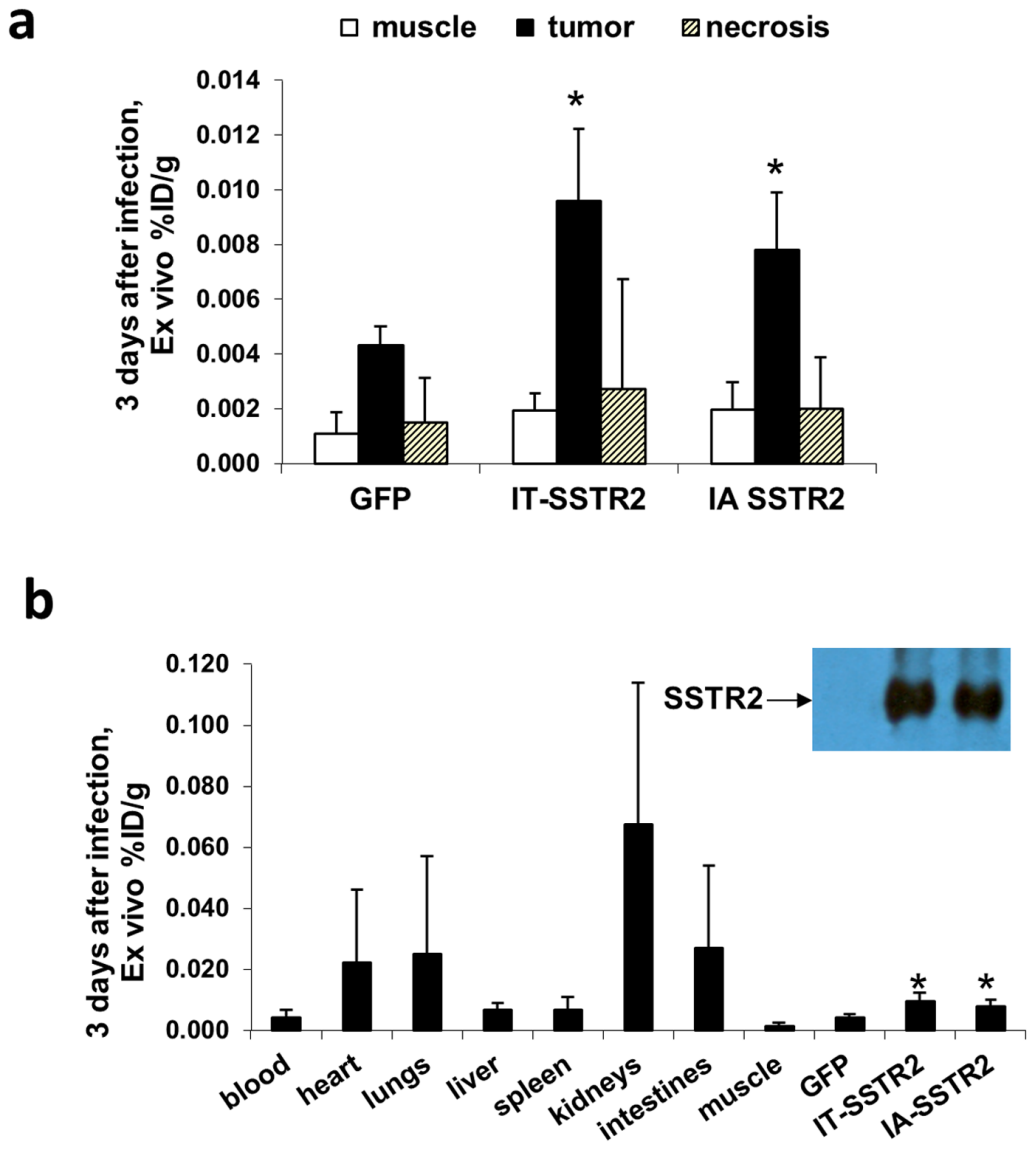
*Ex vivo* analysis of ^111^In-octreotide biodistribution in VX2 tumors 3 days after adenoviral infection. (a) Graph showing significantly higher biodistribution in tumors infected with Ad-CMV-HA-SSTR2 after IA and IT administration compared to tumors infected with control Ad-CMV-GFP (*, p<0.01 for IA, n = 6 and p<0.02 for IT, n = 6). The %ID/g was higher in viable tumor tissue compared to areas of tumor necrosis (p<0.001 for IA and p<0.01 for IT, n = 6) or muscle (p<.001 for IA and p<0.001 for IT, n = 6). (b) Graph showing *ex vivo* tissue biodistribution of ^111^In-octreotide in multiple organs of rabbits bearing VX2 tumors infected *in vivo* with Ad-CMV-HA-SSTR2 or control virus. As in (a), increased uptake was seen in tumors infected with Ad-CMV-HA-SSTR2 by intra-arterial and intra-tumoral routes compared to tumors infected by intra-tumoral route with control Ad-CMV-GFP and this was further confirmed by Western blotting.

Ex vivo evaluation of the radiotracer biodistribution demonstrated significantly greater %ID/g in tumors infected with Ad-CMV-HA-SSTR2 by both the IA and IT routes as compared to tumors infected with control Ad-CMV-GFP virus (p<0.01 for IA, n = 6; and p<0.02 for IT, n = 6; Fig. 2A) and there was no difference in expression by tumor directed IA or IT routes of Ad-CMV-HA-SSTR2 delivery. Moreover, this was further confirmed by Western blotting (Fig. 2B) with clear, similar HA-SSTR2 bands seen by IA or IT routes routes of Ad-CMV-HA-SSTR2 delivery and background noted with control virus. Of note, background uptake was seen in areas of necrosis (Fig. 2A). As expected, the kidneys, which are the primary sites of radioligand excretion, exhibited the greatest %ID/g (Fig. 2B). Excretion is also known to occur via the liver to intestines and background uptake in the spleen is commonly seen clinically.

### Longitudinal Assessment of Reporter Gene (Ad-CMV-HA-SSTR2) Expression after *In Vivo* Gene Transfer

**Figure 3:**
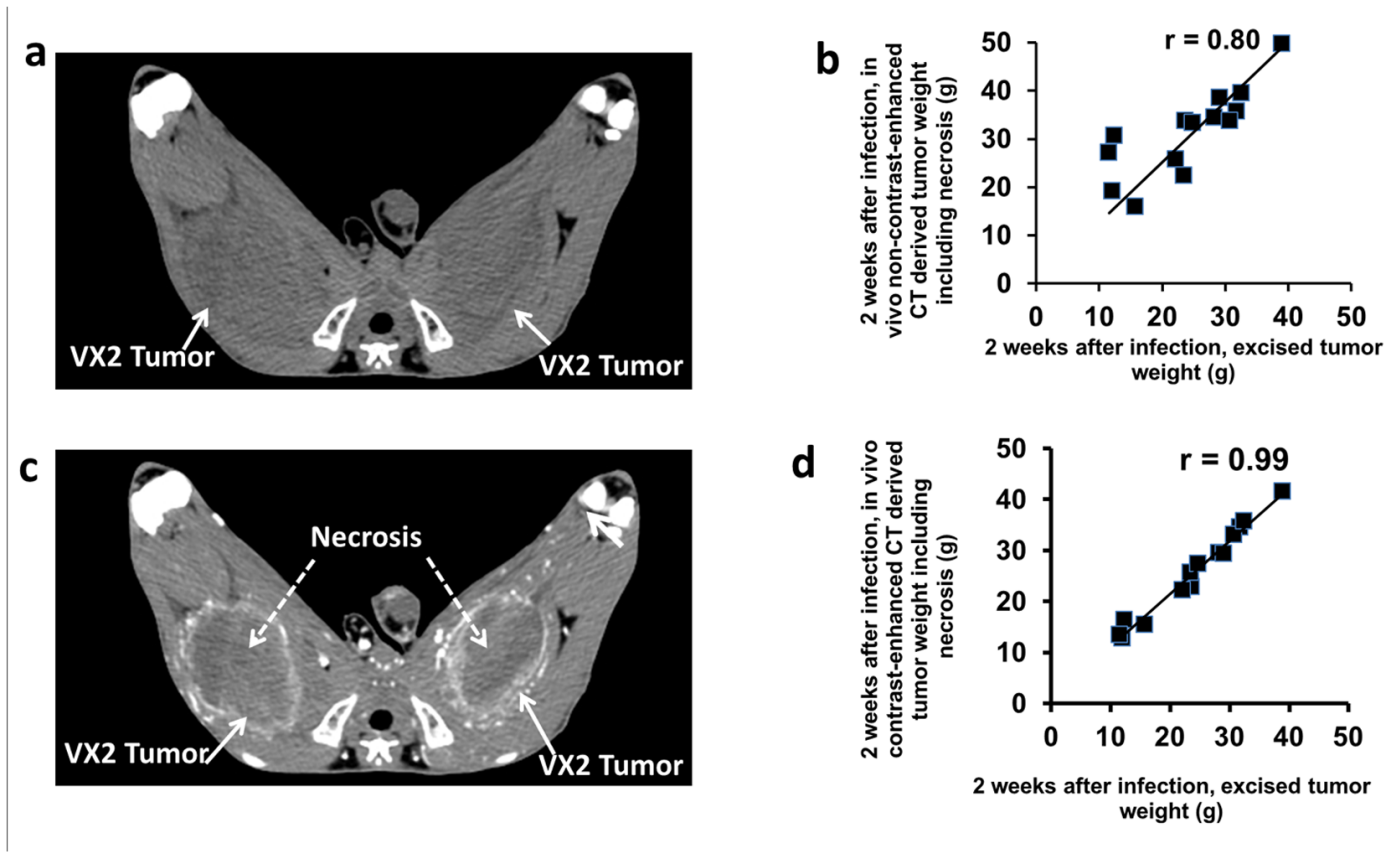
Representative transverse *in vivo* CT images of rabbit hind leg VX2 tumors. (a) Unenhanced (without contrast) CT image acquired 24 days after VX2 inoculation in both thighs. Tumors appear as areas of low attenuation (arrows) and are relatively difficult to visualize. (b) Graph shows that weight derived from unenhanced CT images acquired 24 days after VX2 inoculation correlates well with weight derived from excised tumors (r = 0.80, p<0.001, n = 14). (c) Contrast enhanced CT image 24 days after VX2 inoculation in both thighs showing well-define peripheral rim enhancement of each tumor (solid white arrows). Areas of non-enhancing necrosis (central low attenuation material) are seen inside the tumor. (d) Graph shows that weight derived (with necrosis) from contrast-enhanced CT images acquired 24 days after VX2 inoculation correlates highly with weight derived from excised tumors (r = 0.99, p<0.001, n = 14).

The evaluation of longitudinal gene expression at 5 days and 2 weeks after adenoviral infection was performed using VX2 tumors growing in the rabbit muscles. Representative *in vivo* CT images of VX2 tumors were obtained without and with contrast enhancement (Fig. 3). Tumor weights derived from *in vivo* unenhanced CT imaging (Fig. 3A, day 24 post tumor inoculation) correlated well with the weights of excised tumors (r = 0.80, p = 0.03, n = 14; Fig. 3B). Contrast enhanced imaging at day 24 post inoculation demonstrated peripheral rim enhancement of live tumor and lack of enhancement of the necrotic center (Fig. 3C). Weights derived from contrast-enhanced CT imaging correlated highly with that derived from excised tumor (r = 0.99, P<0.0001, n = 14; Fig. 3D).

**Figure 4:**
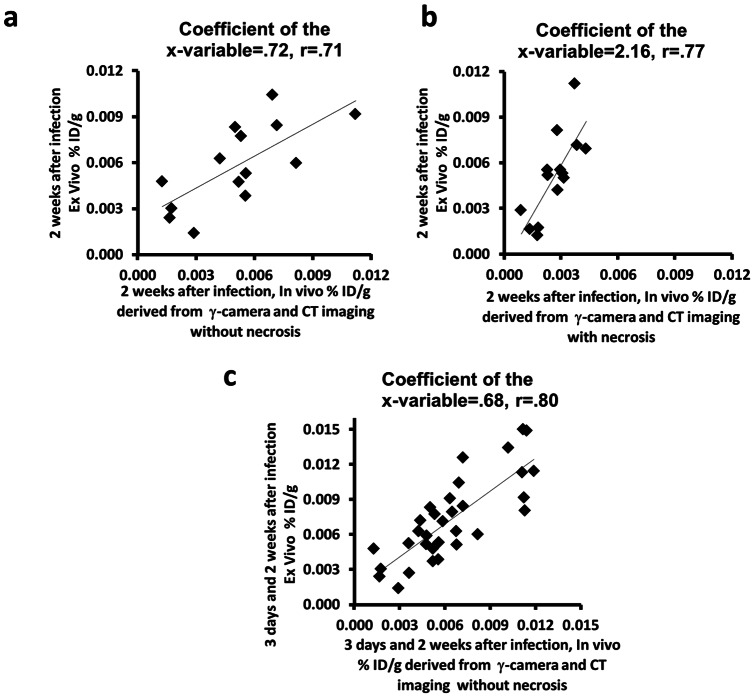
Longitudinal imaging data in VX2 tumors infected with Ad-CMV-HA-SSTR2. (a and c) Representative *in vivo* gamma camera planar images of VX2 tumors obtained at 5 days (a) and 2 weeks (c) after infection with Ad-CMV-HA-SSTR2 (S =  source of ^111^In for positioning, i and ii are two different representative rabbits). (b and d) *In vivo* imaging derived biodistribution of ^111^In-octreotide normalized to tumor weight (%ID/g) calculated with and without necrosis at 5 days (b) and 2 weeks (d) after infection with Ad-CMV-HA-SSTR2. Tumors infected with Ad-CMV-HA-SSTR2 by either IA or IT delivery consistently showed significantly higher levels of the radioligand uptake compared to tumors infected with control Ad-CMV-GFP (*, p<0.05 compared with GFP). (d) % ID/g without necrosis was significantly higher than % ID/g with necrosis in tumors infected with Ad-CMV-HA-SSTR2 in both IA and IT (#, p<0.01 for IA, n = 6 and p<0.01 for IT, n = 4) groups.

Planar imaging was performed 5 days after virus administration (Fig. 4A(i)). Consistent with the results above, tumors infected with Ad-CMV-HA-SSTR2 virus by either the IA or IT routes had similar uptake of ^111^In-octreotide, and this was significantly greater than tumors infected with control (p<0.02 for IA, n = 6 IA, n = 4 GFP; p<0.03 for IT, n = 4, n = 4 GFP; Fig. 4A (ii) and Fig. 4B). Similar findings were noted when the *in vivo* biodistribution was normalized for necrosis (p = 0.01 for IA, n = 6, p = 0.03 for IT, n = 4; Fig. 4B). As in the prior experiment, there was no significant difference between IA and IT delivery at 5 days; similarly, no such difference was seen between with and without necrosis (Fig. 4B).

A second dose of ^111^In-octreotide was given and planar imaging (Fig. 4C) and *in vivo* biodistribution analysis of the tumors was performed of the same rabbits 2 weeks after virus injection. Significantly greater uptake was seen in tumors infected with Ad-CMV-HA-SSTR2 administered by either the IA or IT routes (Fig. 4C(i)) as compared to the tumors infected with control Ad-CMV-GFP virus (p<0.01 for IA, n = 6; p<0.01 for IT, n = 4; Fig. 4C(ii) and Fig. 4D). Again, no difference was noted between the Ad-CMV-HA-SSTR2 IA and IT delivery groups. When the amount of ^111^In-octreotide biodistribution was normalized to the CT-determined tumor weight (% ID/g) without necrosis, Ad-CMV-HA-SSTR2 gene expression remained significantly greater than Ad-CMV-GFP control (p<0.02 for IA, n = 6 and p = 0.01 for IT, n = 4; Fig. 4D). There was a significant difference between with and without necrosis at 2 weeks for both IA (p<0.01, n = 6; Fig. 4D) and IT (p<0.01, n = 4; Fig. 4D) delivery. No such difference was observed with the control Ad-CMV-GFP virus.

**Figure 5:**
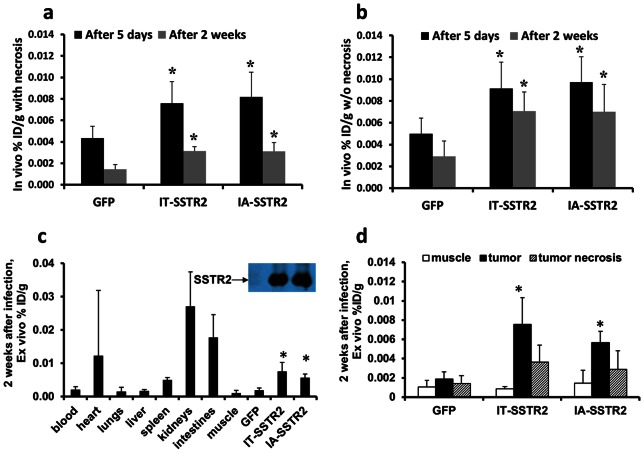
*In vivo* and *ex vivo* ^111^In-octreotide biodistribution. (a and b) *In vivo* radioligand biodistribution normalized to tumor weight (%ID/g) calculated with (a) and without (b) necrosis at 5 days and 2 weeks after infection with Ad-CMV-HA-SSTR2 or control Ad-CMV-GFP (*, p<0.05 compared with GFP). Normalizing for necrosis demonstrates that the apparent loss of expression at the later 2 week time point (compared with 5 days) post infection is less when the confounding variable of necrosis is removed. (c) Graph showing *ex vivo* tissue biodistribution of ^111^In-octreotide in multiple organs in rabbits bearing VX2 tumors 2 weeks post virus infection. Significantly greater uptake was seen in tumors infected with Ad-CMV-HA-SSTR2 by IA or IT routes compared to tumors infected with control Ad-CMV-GFP (c and d: *, p<0.001 for IA, n = 6; p<0.01 for IT, n = 4) and this is confirmed by Western blotting. (d) Graph showing the %ID/g was significantly higher in viable tumor tissue infected with Ad-CMV-HA-SSTR2 compared to tumor necrosis (p<0.01 for IA, n = 6, p<0.02 for IT, n = 4) or muscle (p<0.001 for IA, n = 6, p<0.02 for IT, n = 4) in tumors infected by Ad-CMV-HA-SSTR2, but not control Ad-CMV-GFP.

Expression appeared to wane with time (Fig. 5A); however, the apparent loss of expression at 2 weeks was less when the confounding variable of necrosis was removed (Fig. 5B). The removal of necrosis via the CT images resulted in a significantly greater biodistribution (% ID/g) compared to that calculated from CT-derived tumor weight with necrosis (Fig. 4D).

*Ex vivo* evaluation of the radiotracer biodistribution at 2 weeks confirmed that tumors infected with Ad-CMV-HA-SSTR2 by both IA and IT routes had similar uptake compared to each other, but significantly greater uptake compared to the tumors infected with the control virus (p<0.001 for IA, n = 6; p<0.01 for IT, n = 4; Figs. 5C and D). This was further confirmed by Western blotting (Fig. 5C); clear, similar HA-SSTR2 bands were seen by IA and IT tumor directed delivery routes of Ad-CMV-HA-SSTR2, whereas, background was noted with control virus. Again, only background uptake was noted in areas of tumor necrosis (Fig. 5D). As expected, the kidneys exhibited the highest level of ^111^In-octreotide uptake (Fig. 5C).

**Figure 6:**
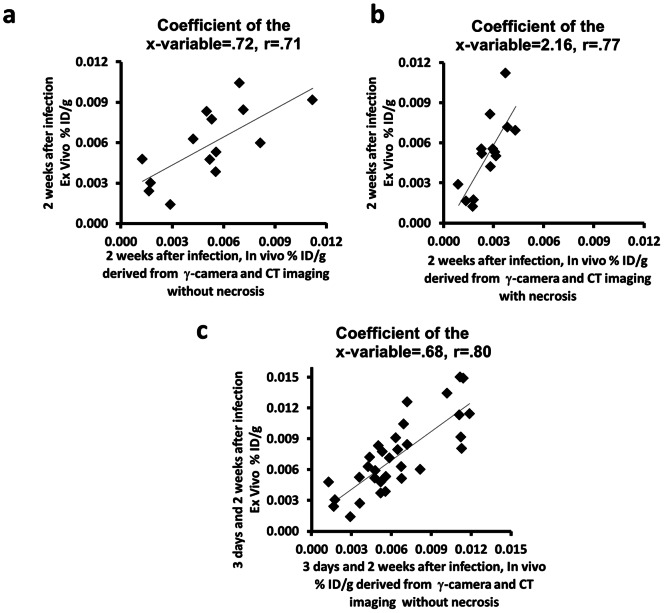
Graphs showing the correlation of image-based *in vivo* analysis with *ex vivo* biodistribution of ^111^In-octreotide in VX2 tumors after *in vivo* **gene transfer.** (a and b) Uptake of radioligand administered 2 weeks after adenovirus normalized to CT-derived tumor weight calculated without and with necrosis. Percentage of injected dose per gram (%ID/g) of excised VX2 tumors correlated with that derived from planar and CT images after 2 weeks of adenoviral infection (a) without necrosis (r = 0.71, p<0.01, Coefficient of the x-variable = .72, n = 14) and (b) with necrosis (r = 0.77, p<0.01, Coefficient of the x-variable = 2.16, n = 14). (c) *In vivo* evaluation of the radiotracer uptake (%ID/g) in viable tumor (without necrosis) derived from planar and CT images after 3 days and 2 weeks of adenoviral infection, correlated with excised VX2 tumors (r = 0.80, p<0.001, Coefficient of the x-variable = 0.68, n = 32).

Image-based noninvasive *in vivo* biodistribution assessment using functional planar and anatomic CT images without necrosis correlated with *ex vivo* assessment at 2 weeks after adenovirus infection (r = 0.71, p<0.01, coefficient of the x-variable = 0.72; Fig. 6A). The correlation with necrosis was 0.77 (p<0.01, n = 14), but the slope of the correlation (coefficient of the x-variable) was 2.16 (Fig. 6B). At 2 weeks, the slope of the correlation without necrosis corresponds better with the ex vivo data and was significantly different from the slope of the correlation with necrosis (p<0.01, n = 14; Fig. 6A and 6B), indicating that performing the measurement excluding necrosis is more representative of gene expression in viable tissue. As noted above, calculated Ad-CMV-HA-SSTR2 gene expression was significantly greater without necrosis than with necrosis at 2 weeks (Fig. 4D). Thus, *in vivo* radiotracer uptake (% ID/g) in viable tumor was calculated without necrosis from region of interest analysis of the planar and CT images obtained 3 days and 2 weeks after adenoviral infection. This combined data correlated with radioactivity associated with excised tumors and the coefficient of the X-variable suggested good correspondence between the in vivo and ex vivo data (r = 0.80, p<0.001, coefficient of the x-variable = 0.68, n = 32; Fig. 6C).

## Discussion

Large animal models bridge the gap between *in vivo* research in rodents and translation to clinical application of novel therapeutic concepts in patients. To the best of our knowledge, this study represents the first report of noninvasive *in vivo* imaging to quantify gene expression in a large animal model using clinical instrumentation incorporating morphologic assessment. It is the first to quantitatively demonstrate the importance of morphology in quantifying expression and we suggest that this finding may be applied to not only this reporter, but others as well. Findings indicate the need to remove necrosis when quantifying gene expression. Furthermore, this is the first study to compare tumor directed IA versus IT gene delivery and to illustrate SSTR2-based reporter imaging in a large animal model.

Both tumor size and gene expression change over time, due to their natural history or treatment, tumors also undergo morphologic changes such as necrosis. Following gene therapy, areas of the tumor may undergo necrosis due to the therapy's direct effect on tumor cells or vascular supply; necrosis may also occur when the tumor has outgrown its blood supply. Theoretically, these areas of necrosis should not express the reporter gene, thus should not contribute to the functional signal that requires viable cells to express the transgene. Therefore, necrotic areas should be excluded from the assessment of gene expression. Despite this expectation, it is also possible that areas of necrosis would non-specifically pool the radiopharmaceutical. Our data demonstrate that the latter is minimal in our SSTR2-based imaging system and that when quantifying expression these necrotic areas should be removed. Functional imaging alone presents a challenge in quantifying gene expression since it has difficulty defining tumor boundaries and differentiating the necrotic areas. Anatomic *in vivo* imaging, such as CT or MR [11], [14]–[16], can measure tumor size and characterize morphology [14], [18], [30], [34].

Quantification of HA-SSTR2 expression after *in vivo* gene transfer in a mouse model has previously been demonstrated using gamma-camera and small animal cognate MR machines [17], [18], [30]. Unenhanced CT imaging has poor soft-tissue resolution for small animal imaging [17], making it difficult to assess morphology in rodent models. In addition, tumors of only approximately 1.5 cm can be grown in a mouse, limiting the evaluation of the contribution of tumor necrosis. In the current study, a prospective solution to this problem was found in the large animal model, which enabled study of larger tumors that underwent necrosis and also enabled use of clinical machines. On contrast-enhanced CT, necrosis was identified as areas in tumors that did not enhance, thus, could be removed to calculate live tumor size or weight.

Such normalization is important in accurately measuring gene expression; for example, normalizing for necrosis resulted in increased tumoral SSTR2 expression calculated in vivo using CT and gamma camera imaging. Furthermore, the *in vivo* imaging data correlated with the *ex vivo* results that were generated from excised tumor tissue. Thus, our findings suggest that tumor necrosis confounds the quantification of reporter gene expression and should be excluded from analysis.

The large animal model also allowed selective vascular access approximating humans, thereby, enabled comparison of expression after tumor directed IA or IT gene delivery. Intra-arterial delivery is standard clinical practice, for example, in interventional radiology and cardiology suites. It is commonly used for vascular procedures such as angioplasty and stenting, as well as for drug delivery such as chemo-embolization of tumors. Using a catheter to deliver a gene is a very minor modification and has been performed clinically [35]–[37].

Vascular access is difficult in murine models due to smaller vessel size. Previous studies have investigated IA and IT delivery methods individually, but not in combination. For example, Kwon *et al*. performed adenoviral intra-arterial delivery in a rat model and demonstrated lac Z expression histologically [38]. In comparison, Chen *et al*. [39] delivered Ad-SSTR2-EGFP IT in a mouse model and detected expression by *in vivo* nuclear and optical imaging; however, *in vivo* quantification was not evaluated. For the first time, we were able to directly compare IA and IT gene transfer *in vivo* within a large animal model and quantify gene expression. We found similar levels of HA-SSTR2 expression after tumor directed IA or IT adenovirus gene delivery. This was consistent in the *in vivo* biodistribution analyses and confirmed by *ex vivo* biodistribution analyses and by Western blotting analyses. We speculate that vascular manipulation in future studies may improve gene delivery and expression after IA delivery.

In the present study, longitudinally, five days after either IA or IT delivery of Ad-CMV-HA-SSTR2, high levels of tumoral gene expression were detected. Two weeks later, expression was still visualized; however, both tumor size and necrosis had increased. Removing necrosis resulted in significantly greater calculated expression in remaining viable tumor, emphasizing the importance of morphology. Thus, the longitudinal change in expression could be assessed and normalized for viable tumor.

SSTR2-based gene expression may be detected noninvasively using ^111^In-octreotide, a clinically available radiopharmaceutical. Previous *in vivo* imaging studies have used SSTR2-based reporter systems in rodent models and small animal cognates of imaging instruments [11], [14], [16], [17], [18], [30]. For the first time, we used a SSTR2-based reporter in a large animal model and demonstrated noninvasive quantification of gene expression using clinical machines. Our findings support the use of SSTR2-based reporters. Novel reporters, such as signaling deficient-SSTR2, should find utility since they are limited in their effects on normal cell signaling and cellular function [16]. Moreover, linking SSTR2 to a therapeutic gene may help generate novel treatment options that can be followed noninvasively. The fact that carcinoid tumors, one of the very rare tumors that naturally overexpress SSTR2, have been imaged clinically for many years using ^111^In-octreotide supports the current data that imaging SSTR2-based reporters will function in patients. Methods to noninvasively monitor gene transfer using SSTR2-based reporters should provide valuable data for applications such as vector development, gene therapy, and assessment of promoter function. Adenoviruses may be used to deliver gene therapy for cancer as well as other diseases. The SSTR2-reporter cassette is amenable insertion into multiple vectors such as adeno-associated virus, lentivirus, retrovirus to follow their expression pattern; and, it should be possible to link it to various therapeutic genes so that expression of the therapeutic gene correlates with expression of the SSTR2-based reporter. The ability to longitudinally and noninvasively evaluate expression in tumors will further enable personalization of gene therapy.

In summary, our investigation demonstrates that high transgene expression can be achieved by both intra-arterial and direct intra-tumoral delivery in a large animal tumor model. For functional imaging, an FDA approved radiopharmaceutical was used. We found that areas of necrosis do not pool this radiotracer, but instead had background levels of uptake. Morphologic analysis enabled removal of areas of necrosis, which can confound quantification of reporter gene expression by living cells. Thus, using clinical machines, noninvasive *in vivo* functional imaging and morphological evaluation of anatomic imaging can be used to quantify the longitudinal expression of a SSTR2-based reporter gene in tumors after *in vivo* gene transfer. These results should contribute to the translation of SSTR2-based reporter systems into clinical practice.
