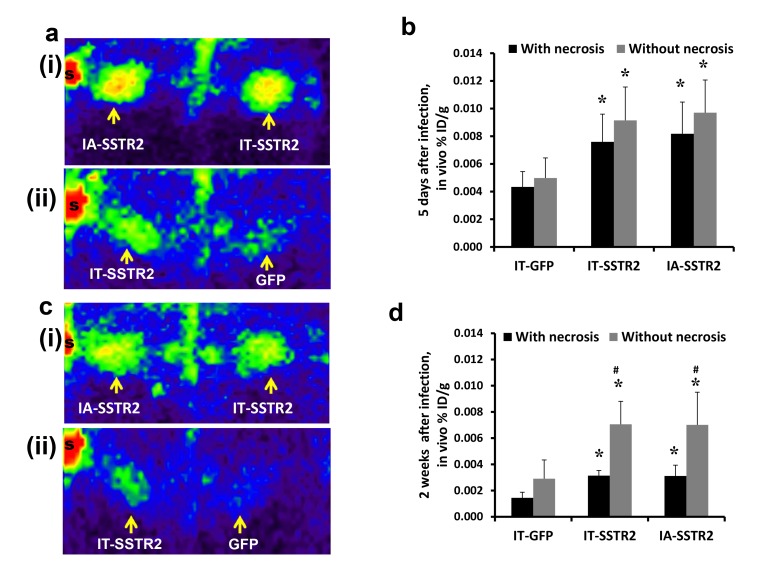# Correction: Noninvasive Assessment of Gene Transfer and Expression by *In Vivo* Functional and Morphologic Imaging in a Rabbit Tumor Model

**DOI:** 10.1371/annotation/d6e7cf90-9e67-4d3b-8a2a-40d57be92bce

**Published:** 2013-10-04

**Authors:** Murali K. Ravoori, Lin Han, Sheela P. Singh, Katherine Dixon, Jyoti Duggal, Ping Liu, Rajesh Uthamanthil, Sanjay Gupta, Kenneth C. Wright, Vikas Kundra

The image for Figure 4 has been mistakenly replaced with the image for Figure 6. The correct image for Figure 4 can be found here: 

**Figure:**